# Innovative Applications of Robotic Surgery: Renal Allograft and Autologous Transplantation

**DOI:** 10.12688/f1000research.7343.1

**Published:** 2016-01-22

**Authors:** Jason Lee, Michael Ordon

**Affiliations:** 1 Department of Surgery, University of Toronto, Toronto, M5C2T2, Canada; 2 Li Ka Shing Knowledge Institute, St Michael's Hospital, Toronto, M5B 1W8, Canada

**Keywords:** Kidney Transplantation, Renal Allograft Transplantation, Robotic Renal Transplantation, Renal Autologous Transplantation, Robotic Surgery

## Abstract

Robotic surgery has enabled surgeons to offer more patients a minimally invasive surgical option in the management of their complex diseases. While renal transplantation is associated with significant improvements in quantity and quality of life for most end-stage renal disease (ESRD) patients, it is also not devoid of its surgical risks and potential morbidities. Robotic-assisted kidney transplantation is a recently described, innovative application of the robotic surgery platform, and early experiences suggest that it is associated with comparable graft function and lower rates of complications.

Urinary tract obstruction, though less common than ESRD, can be a serious threat to renal function. Severe ureteric stricture disease can represent a clinically complex problem requiring major reconstructive surgery. Completely intra-corporeal robotic renal auto-transplantation is another innovative application of the robotic surgery platform and represents a significant advancement in urologic surgery. Initial reports of this procedure demonstrate safety, feasibility, and excellent renal function outcomes.

Robotic-assisted laparoscopic surgery has gained widespread adoption globally within the field of urology. Technologic advancements have led to improved surgical dexterity and vision and, combined with a more facile learning curve than pure laparoscopic surgery, the robotic surgery platform has enabled more surgeons to perform minimally invasive surgical (MIS) procedures. Moreover, not only has robotic surgery afforded more patients access to a MIS option, it has also enabled urologists to perform extremely complex extirpative and reconstructive procedures, traditionally performed in an open fashion due to their technically challenging nature, with a minimally invasive approach.

The first successful kidney transplant was reported in 1956 ^1^ and since that time renal transplantation has changed the lives of millions of patients with end-stage renal disease (ESRD) through improved quantity as well as quality of life ^2^. With the success of living donor renal transplantation programs, outcomes for patients with ESRD have improved even further.

Unlike healthy living donors, most kidney transplant recipients are, by the very nature of their disease, high-risk surgical candidates. While the long-term benefits of renal transplantation have been clearly documented ^3– 5^, there are tangible peri-operative risks for ESRD patients undergoing transplantation that need to be weighed against these long-term benefits.

The advantages of MIS have been well documented in the literature and include less post-operative pain, quicker recovery time, lower rates of incisional complications, less blood loss, and better cosmesis ^6– 8^. Level 1 evidence supports a minimally invasive approach for donor nephrectomy ^9^ in a patient population that is healthy and highly screened and selected. But for the high-risk ESRD recipients, for whom the peri-operative advantages of MIS would be evermore beneficial, a more morbid open surgical approach to renal transplantation still remains the gold standard.

As robotic surgery has become more pervasive and mainstream, and with improved experience and training, it has allowed highly skilled and innovative surgeons to now offer MIS options for these higher-risk ESRD patients.

## Robotic renal allograft transplantation

The first reported use of robotics to perform a kidney transplant was in 2002 ^10^; however, this was in essence a hybrid operation whereby the da Vinci® (Intuitive Surgical Inc, USA) robotic surgical platform was used to perform a conventional open deceased donor kidney transplant through a large open incision.

The first true robotic-assisted laparoscopic kidney transplant (RAKT) was reported by the group from University of Illinois at Chicago in 2010 ^11^. Using the da Vinci® robot, Giulianotti *et al.* performed a deceased donor RAKT utilizing a 7 cm peri-umbilical incision through which the graft was introduced into the peritoneum. Total operative time was 223 minutes, with warm ischemia time of 50 minutes. The following year, Boggi *et al.* published the first successful European RAKT, but described a slightly different technique ^12^. Rather than a peri-umbilical incision, the authors utilized a 7 cm Pfannenstiel incision. While the vascular anastomoses were performed entirely robotically, the ureterovesical anastomosis was performed in an open fashion through the Pfannenstiel incision. Total operative time was 154 minutes, with 51 minutes of warm ischemia. In 2015, Doumerc *et al.* described another novel approach to RAKT, in which they utilized a vaginal incision to introduce the renal graft into the peritoneum, transvaginally, inside a sterile Endobag ^13^. Similar to the other case reports, mean operative time was 200 minutes and anastomotic time was 55 minutes. While only feasible in female ESRD recipients, this novel transvaginal technique eliminates the need for a larger abdominal incision, further accentuating the minimally invasive nature of RAKT and thereby perhaps further decreasing the morbidity of renal transplantation surgery. Building on their novel technique, later that year, Doumerc and Sallusto reported the first pure robot-assisted approach to living donor kidney transplantation utilizing the transvaginal technique for both the donor and the recipient surgeries ^14^.

While these pioneering surgeons demonstrated the safety and feasibility of this innovative procedure, the reported RAKT techniques did not involve intra-corporeal cold perfusion of the graft. In addition, these early reports had longer warm ischemic times than commonly seen with open kidney transplantation. How this slight increase in warm ischemia truly impacts long-term graft function is yet unknown, but clearly delineates an opportunity for improvement.

In 2014, two separate publications from the same authorship group reported on a RAKT case series that utilized a new technique allowing for intracorporeal regional hypothermia of the graft ^15, 16^. The authors used a peri-umbilical incision as well, but described the use of a novel gauze-jacket filled with ice-slush. This served to minimize warm ischemia and also allowed for atraumatic handling of the graft. Additional ice-slush was introduced into the peritoneum to cover the graft once it had been placed into the peritoneum. Utilizing this technique, the mean operative time in the case series was 214 minutes, with a mean “warm” ischemia time of 47 minutes ^15^. The authors cited that while there was clear evidence for the feasibility and safety of this innovative procedure, comparative studies were still required to determine the cost-effectiveness of RAKT over conventional open kidney transplantation.

In line with existing comparative literature evaluating laparoscopic and open surgery, initial experiences at various institutions around the world have now demonstrated lower complication rates for RAKT in comparison to similar open renal transplant cohorts ^15, 17, 18^. With comparative ischemia times and the ability to cool the graft intracorporeally, graft function outcomes also seem to be at least equivalent to traditional open renal transplantation ^15, 17^.

Among a cohort of morbidly obese ESRD patients, Oberholzer *et al.* demonstrated that RAKT was associated with better outcomes in comparison to conventional open kidney transplantation. The authors pointed out that with improved outcomes after RAKT in the morbidly obese patient population, it may result in increased access to life-saving transplantation surgery for patients who may have otherwise been deemed unsuitable candidates due to their increased peri-operative risks ^17^.

These initial RAKT cohorts still represent a small sample size, and as such comparative assessment with larger cohorts will be necessary in time to further support the initial findings, which are very encouraging.

## Robotic renal autologous transplantation

One of the most common criticisms regarding the role of RAKT is the fact that an open incision, at least the size of the graft, is required regardless in order to introduce the allograft kidney into the peritoneal space, so why not simply use a slightly larger incision and do the surgery in a more conventional manner. RAKT supporters argue that any minimization of incision length, particularly in the immunosuppressed, ESRD patient population, can decrease the not-insignificant surgical incision-related complication rates seen in these patients. This is particularly true among obese transplant recipients, who are prone to much higher rates of incisional complications ^17^.

Renal autologous transplantation (ReATx) was first reported in 1963 and represents a viable option in the management of long or severe upper ureteric strictures ^19^. While much less commonly performed than allograft renal transplantation, ReATx is a definitive surgical option that allows for the preservation of renal function, whether imperative or elective, while reconstituting normal urinary drainage. The morbidity of ReATx is not inconsequential, however, as it involves two very distinct and complex procedures.

The laparoscopic approach to the management of many different urologic diseases has now become commonplace. With this, the morbidity of ReATx has significantly improved as well and, currently, the most common approach to ReATx is laparoscopic donor nephrectomy followed by open ReATx ^20, 21^. This approach has demonstrated excellent outcomes and is considered by many to be the gold standard approach.

Unlike deceased donor or living donor renal transplantation surgery, for patients deemed suitable for ReATx, the allograft kidney is already located intra-corporeally. As such, if one were to be able to perform both distinct stages of the ReATx (i.e. donor nephrectomy and auto-transplantation), utilizing a MIS technique while maintaining the allograft intra-corporeally, the morbidity associated with a large surgical incision would be avoided completely. The ability to do this adds extreme technical complexity and would necessitate not only intra-corporeal preparation of the graft but completely intra-corporeal perfusion, and hypothermia as well.

Taking their experience with RAKT one step further, Abaza and colleagues reported the first ever completely intra-corporeal robotic-assisted ReATx surgery in 2014 ^22^. This truly innovative application of the robotic surgery platform allowed for the management of severe ureteric stricture disease without the allograft ever having to be removed from the patient. Total operative time was 425 minutes and total ischemic time was 127 minutes. While this pioneering report demonstrated the safety and feasibility of robotic-assisted ReATx, the described technique was associated with longer ischemia than one would encounter with the conventional approach to ReATx: laparoscopic donor nephrectomy, *ex vivo* preparation, and open auto-transplantation ^9, 23^.

Building on their seminal work, we reported the first completely intra-corporeal robotic-assisted ReATx in Canada ^24^ but modified the technique utilized by Abaza and colleagues ^22^ in an attempt to minimize renal ischemia. Similar to their described technique, intra-corporeal renal perfusion with cooled HTK and normal saline solution was initiated immediately after donor nephrectomy using a perfusion cannula inserted through a 12 mm assistant port. By altering the technique from a two-stage to a three-stage procedure, however, we were able to decrease the total operative time to 390 minutes and, more significantly, we were able to complete the surgery with only 79 minutes of ischemia (4 minutes of warm ischemia, 48 minutes of cold ischemia, and 27 minutes of re-warming time), which is more comparable to what is seen with conventional ReATx surgery.

While these two initial reports have demonstrated the safety and feasibility of completely intra-corporeal robotic ReATx, both case reports involved kidneys with relatively straightforward renal vasculature. With any increased complexity (e.g. two or more renal arteries), vascular reconstruction without removal of the kidney *ex vivo* would necessitate significant alterations in technique. We are currently working on developing such techniques that would allow for the allograft to remain intra-corporeally, while simultaneously minimizing renal ischemia during vascular reconstruction and auto-transplantation.

## Conclusions

Robotic surgery has enabled surgeons to offer more patients a minimally invasive surgical option in the management of their complex diseases. Robotic-assisted kidney transplantation and completely intra-corporeal robotic renal auto-transplantation are recent innovative applications of the robotic surgery platform and represent significant advancements in urologic surgery. These novel applications of robotic surgery will hopefully result in improved patient outcomes while simultaneously achieving lower patient morbidity.
